# TTAS: Trusted Token Authentication Service of Securing SCADA Network in Energy Management System for Industrial Internet of Things

**DOI:** 10.3390/s21082685

**Published:** 2021-04-11

**Authors:** Yu-Sheng Yang, Shih-Hsiung Lee, Wei-Che Chen, Chu-Sing Yang, Yuen-Min Huang, Ting-Wei Hou

**Affiliations:** 1 Department of Engineering Science, National Cheng Kung University, Tainan City 701, Taiwan; n98991108@mail.ncku.edu.tw (Y.-S.Y.); huang@mail.ncku.edu.tw (Y.-M.H.); houtw@mail.ncku.edu.tw (T.-W.H.); 2 Department of Intelligent Commerce, National Kaohsiung University of Science and Technology, Kaohsiung City 824, Taiwan; 3 Institute of Computer and Communication Engineering, National Cheng Kung University, Tainan City 701, Taiwan; q36084214@gs.ncku.edu.tw (W.-C.C.); csyang@ee.ncku.edu.tw (C.-S.Y.)

**Keywords:** authentication, token, SCADA security, Modbus, energy management system, industrial internet of things (IIoT)

## Abstract

The vigorous development of the Industrial Internet of Things brings the advanced connection function of the new generation of industrial automation and control systems. The Supervisory Control and Data Acquisition (SCADA) network is converted into an open and highly interconnected network, where the equipment connections between industrial electronic devices are integrated with a SCADA system through a Modbus protocol. As SCADA and Modbus are easily used for control and monitoring, the interconnection and operational efficiency between systems are highly improved; however, such connectivity inevitably exposes the system to the open network environment. There are many network security threats and vulnerabilities in a SCADA network system. Especially in the era of the Industrial Internet of Things, any security vulnerability of an industrial system may cause serious property losses. Therefore, this paper proposes an encryption and verification mechanism based on the trusted token authentication service and Transport Layer Security (TLS) protocol to prevent attackers from physical attacks. Experimentally, this paper deployed and verified the system in an actual field of energy management system. According to the experimental results, the security defense architecture proposed in this paper can effectively improve security and is compatible with the actual field system.

## 1. Introduction

With the development and maturity of the Internet of Things technology, a large number of related technologies are being used to realize intelligent manufacturing in the field of industrial automation and information analysis [1]. The application of the Internet of Things in industrial markets is called the Industrial Internet of Things (IIoT) [2], which enables the constant development of new industrial controls, industrial automation, job service processes, production line data analysis, and new business models. Such technological evolution is called the Fourth Industrial Revolution or Industry 4.0 [3]. The increasingly vigorous development of related technologies in Industrial Control Systems (ICS) [4] promotes the national infrastructure, as well as industrial, agricultural, and manufacturing industries, in order to obtain momentum for digital transformation. At present, there are many successful cases in many related studies and practical cases, such as the application of the Supervisory Control and Data Acquisition (SCADA) [5] of ICS, which is applied in combination with the Programmable Logic Controller (PLC) [6] to realize the reservoir control system [7], and the application of the Distributed Control System (DCS) [8] of ICS to realize temperature regulation systems, as applied in [9] for large commercial buildings. Therefore, the industrial control system has a pivotal position in both economic development and national security. Distributed Network Protocol (DNP) [10] is a communication protocol used between automation components, commonly used in power, water treatment, and other industries. SCADA can use the DNP protocol to communicate with the master station and remote terminal unit. In order to effectively integrate Information Technology (IT) and Operation Technology (OT), the Open Platform Communications Unified Architecture (OPC UA) [11] industrial automation communication standard integrates equipment information and connects to the cloud to achieve cross-platform remote monitoring and robust information security features. In addition, OPC UA supports common industrial communication protocols such as MQTT [12] and Modbus. Moreover, the Energy Management System (EMS) [13], which is also used for monitoring, managing, and controlling systems, has also developed many applications along with the technological improvements of the Industrial Internet of Things, such as the Building Energy Management System (BEMS) [14], which is applied to residences and centered on residents. In addition, demand response (DR) is used to balance the supply and demand of electricity and avoid energy waste [15]. Furthermore, three technologies—the Home Energy Management System (HEMS) [16], the Internet of Things, and big data analysis—are combined to achieve the purposes of collecting and analyzing data, control, and saving energy [17]. EMS has become a mature technology and is widely used in new residences and new buildings. Smart green energy management applies a wide range of technologies, including SCADA, Community Management System (CEMS), BEMS, PLC, and various Internet of Things technologies.

Moreover, as the system architecture mentioned in this paper is applied to the national infrastructure project, the security requirements are particularly strict. Therefore, the defense scheme is quite perfect in the establishment of a firewall, the Intrusion Detection System (IDS) [18], and the Intrusion Prevention System (IPS) [19], which guards against external network attacks; however, there is no defense against the internal network. Therefore, if hackers use a physical man-in-the-middle attack (MITM) [20] on this infrastructure, or manage to connect to the internal network through some method, it may cause serious damage. SCADA has been widely used in various national infrastructures, manufacturing, and industries, such as power grids, smart manufacturing, transportation systems, sewage treatment plants, oil exploitation, etc. Therefore, the security protection abilities of SCADA should be strengthened, such as security and privacy protection mechanisms. The SCADA system is mostly realized by integrating the Modbus industrial communication protocol. However, when Modbus was first proposed, it was used for speed and convenience, and it was only meant to be used on internal networks. As a result, there were serious security vulnerabilities in the Modbus design. The growing demand for automatic industrial control systems has brought SCADA from strictly isolated networks to the highly interconnected Internet [21]; thus, infrastructure is exposed to the risk of information security and may be attacked by hackers at any time, which will result in significant economic losses. However, with the rapid development of intelligent energy facilities (for example, smart grids and microgrids), the authors of [22,23] pointed out that the current open protocol Modbus TCP to deploy smart grids or microgrids can effectively improve the heterogeneity of the required hardware and software components. Modbus TCP currently plays an important role in the system integration of monitoring, operation, and maintenance in industrial applications. Therefore, in order to solve the above problems, this paper proposes an encryption and verification mechanism based on Tokens and the Transport Layer Security (TLS) Protocol to prevent hackers from engaging in physical attacks. The system architecture mainly includes a Trusted Token Authentication Service (TTAS), a Trusted Verification Module (TVM), and an Encrypted Validator (EV). The contribution of this paper is that the proposed system has actually been deployed and verified in the energy management system of a Green Energy Science City in southern Taiwan. Actual usage shows that it can effectively prevent man-in-the-middle attacks by adding encryption and verification mechanisms, and it can also solve the security vulnerabilities of Modbus using plaintext to transfer packets. In addition, by increasing a small amount of computing, transmission, and storage overhead, it avoids causing the control program of the system to be unable to access to the device’s reply in real-time and send requests repeatedly, leading to its failure to obtain device information, and resulting in errors and even system crash. According to the experimental results, the security defense architecture proposed in this paper can effectively improve security and is compatible with the actual field system.

The remainder of this paper is organized as follows. Section 2 summarizes the related research work. Section 3 defines the problems to be solved. Section 4 introduces the TTAS architecture proposed in this paper. Section 5 offers security analysis. Section 6 presents the experimental results. Finally, Section 7 offers conclusions.

## 2. Related Works

This section will introduce related works including the security and authentication of the Internet of Things and TLS protocol.

### 2.1. The Security of the Industrial Internet of Things

The core of the Industrial Internet of Things is the Industrial Control System (ICS). How to protect the reliability and security of ICS systems, such as the SCADA system, the Distributed Control System (DCS), and PLC, has become an important issue. The man-in-the-middle attack is very common in the IIoT environment, and attackers often use Address Resolution Protocol (ARP) spoofing to gain access rights and manipulate router traffic and message flow [24]. Once an attacker has successfully intruded the system, it is possible to implement a replay attack [25], which many cause the system and equipment to fail to operate according to normal procedures. In addition, the combination of control data modification and replay attacks has infinite possibilities and can cause serious damage without proper protection. In order to mitigate related risks, the authors of [4] provided corresponding security countermeasures for the threats and vulnerabilities faced by the ICS system. With the development of the Internet of Things and communication technology, the control system of the IIoT is no longer closed. In [26,27], security indicators and test methods were analyzed and compared for the communication protocols (such as Modbus) and attack threats of industrial control systems. The authors of [21] used the standard vulnerability database and actual cases to describe various types of potential SCADA risks, including ARP spoofing and man-in-the-middle attacks through the TCP/IP protocol. At present, international regulatory bodies are actively revising SCADA protocol standards for vulnerabilities and risks; however, due to the high complexity of IIoT integration, it requires a lot of time to provide a complete protection strategy. The authors of [28] provided comprehensive analysis and classification of the SCADA communication architecture and threats. The authors of [28] pointed out that the main security threats are the use of lightweight private key exchange mechanisms and defenseless service attacks. Therefore, in addition to adopting an encryption mechanism, the establishment of the SCADA defense mechanism is particularly important for the authentication and authorization of access rights. The SCADA system is mostly realized by integrating the Modbus industrial communication protocol; unfortunately, Modbus itself lacks many security mechanisms to protect against vulnerabilities, such as the security vulnerabilities of Modbus using plaintext to transfer packets. The authors of [29] adopted the TLS protocol to improve the confidentiality and security of data transmission. The authors of [30] proposed a Role-Based Access Control (RBAC) model that integrates TLS and X.509v3 certificate protocol to authorize the client on the server and Modbus devices at the same time. RBAC effectively solves the security problems of the Modbus protocol. The authors of [31] provided a Modbus TCP solution integrating trusted platform module (TPM) functions to ensure the correctness of sent messages and effectively resist man-in-the-middle attacks. The authors of [22] used machine learning and artificial intelligent technologies to implement an intrusion detection system by detecting Modbus TCP packets of possible network attacks and abnormality. In addition, industrial equipment interactively communicates with the SCADA system through the Modbus TCP/IP protocol, thus the authentication of the device can be realized on the Modbus TCP side. The authors of [32] proposed an authentication mechanism for industrial sensors through Modbus TCP. In the process of TCP/IP packet transmission, the authentication hash code is generated by intercepting the header fields of the TCP packets, in order to confirm the sensor identity. Therefore, this paper proposes an encryption and verification mechanism based on the trusted token authentication service and Transport Layer Security (TLS) protocol to improve the security of the SCADA system and Modbus protocol.

### 2.2. TLS Protocol

TLS refers to Transport Layer Security, which is based on the Secure Sockets Layer (SSL). TLS provides secure communication between two communication entities, such as web browsers and servers. The connection of TLS itself is safe and reliable, and the transmitted data are encrypted by using the symmetric encryption mechanism. Moreover, each key is uniquely generated by the TLS handshake during each connection. In addition, running the TLS handshake protocol on the basis of the TLS recording protocol allows two communication entities to authenticate each other. During the TLS handshake, the communication entities can use an asymmetric encryption mechanism (such as RSA) to authenticate each other. The TLS 1.3 version was released in August 2018 [33] and includes many security and performance improvements. TLS 1.3 further improved the speed of encrypted connections through TLS false start and Zero Round Trip Time (0-RTT). Moreover, TLS 1.3 removed the obsolete and insecure functions of TLS 1.2, such as SHA-1, AES-CBC, MD5, etc. TLS 1.3 renders the connection communication both faster and safer, meaning attackers cannot easily break this protocol, as no information can be revealed to the attacker through an authenticated connection. In addition, as the attacker cannot be detected by the communication entity in the TLS protocol, the attacker cannot modify the content of communication negotiation. Therefore, this paper uses TLS 1.3 to encrypt Modbus TCP to improve the security of communication.

### 2.3. The Authentication in IoT Environment

The Internet of things represents billions of interconnected devices, which are usually equipped with sensors and communication devices, and the devices access data and transmit commands through a communication protocol. How to validate and authorize permissions for such a large number of devices is a challenge, and authentication is especially important for the IIoT, as unauthenticated malicious devices may cause damage. The authors of [34] sorted a variety of authentication protocols and evaluated the advantages and disadvantages of identity authentication protocols. In the three-layer architecture of the Internet of Things (perception layer, network layer, and application layer), authentication is necessary in the definition of these three layers. The authentication methods on the Internet of Things are mainly divided into hardware-based methods (True Random Number Generator, Physical Unclonable Function, and Trusted Platform Module), token-based methods, and procedure methods (one-way, two-way, and three-way) [34]. In addition, as the Internet of Things has the characteristics of low power consumption and limited storage space and processing capacity, it is quite limited in terms of reliability, computing performance, security, and confidentiality. Therefore, how to design a lightweight authentication protocol has become an important issue. In the hardware-based scheme, Physical Unclonable Function (PFU) initializes the PFU information in the device through the time-based OTP (TOTP) method, and then conducts the lightweight independent mutual authentication protocol [35]. The authors of [36] proposed a PUF-IPA (Identity Preserving Protocol) through a self-checking mechanism to preserve the device identity and to connect the database with the server. PUF-IPA provides an effective solution for security, reliability, and device privacy protection. The authors of [37] proposed a PUF-based method for the first key sharing. It enables new applications for lightweight key sharing protocol. PUF is based on hardware implementation, and its advantages are lightweight and performance. However, there are still many devices in IIoT that only support simple reading, writing, and actions based on requests. The method proposed in this paper is based on software. Although it is inferior to the PUF-based method in terms of performance, it can increase the security without affecting the performance of the current system operation in the integration of the existing actual field. In addition, the method proposed in this paper has better advantages in terms of flexibility and security mechanism upgrade cost compared to hardware-based solutions. The authors of [38] further proposed a lightweight mutual verification and key exchange model for remote devices, which has been applied in IIoT. In the process of device authentication, symmetric and asymmetric key encryption, hash codes, and timestamps were used to effectively prevent replay attack, modification attacks, and man-in-the-middle attacks. The authors of [39] proposed a machine-to-machine (M2M) lightweight mutual authentication mechanism for application to IIoT, which uses only hash and XOR operations. In the token-based scheme, a token has a high degree of flexibility to implement verification and authorization mechanisms. In addition, as the token is issued by a third-party verification center, it can improve reliability and security. Moreover, as tokens have the feature of high privacy, they can prevent sensitive information from being easily disclosed. The authors of [40] proposed a token-based lightweight user authentication method (TBLUA) to enhance the robustness and security strength of authentication. The authors of [41] mentioned that Device Language Message specification (DLMS)/COmpanion Specification for Energy Metering (COSEM) are protocols often used in smart grids for data exchange. In addition, DLMS also supports High-Level Security (HLS) authentication, through mutually challenge exchange, and verification of challenge result between DLMS server and client. The above-mentioned results show that low computing cost, low communication and storage overhead, the realization of identity authentication, and attack resistance are the key issues to achieve IIoT security.

## 3. Problem Definition

**Figure 1:**
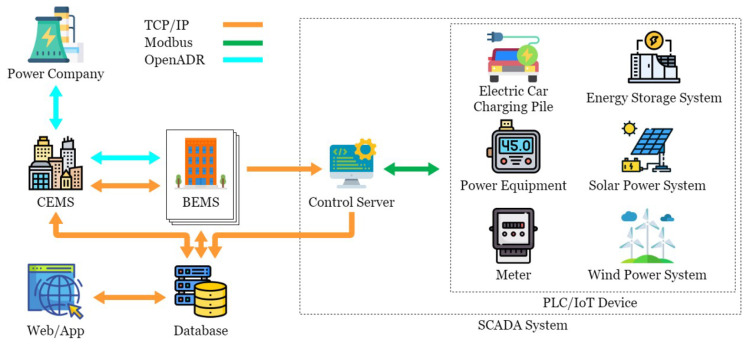
Energy management system in the Green Energy Science City in southern Taiwan.

**Figure 2:**
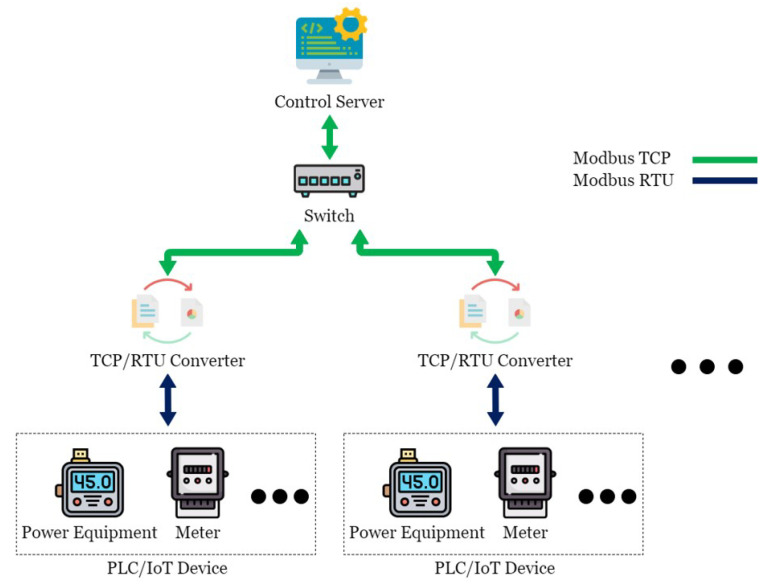
SCADA system in the Green Energy Science City in southern Taiwan.

This paper conducted experimental testing in an actual energy management system in the Green Energy Science City in southern Taiwan. This energy management system is mainly composed of a Taiwan Power Company system, a community energy management system, a building energy management system, and the SCADA system, as shown in Figure 1. OpenMUC [42] is used to implement the SCADA system. Open Automated Demand Response (OpenADR) is an open-source software for energy management research and standard development [43], which is used to send messages and signals, and is committed to establishing general standards for smart grids. OpenADR provides demand response solutions to users. In addition to participating or not participating, users do not actively provide electric energy to participate in the electric energy market in real-time two-way channels. In addition, in order to avoid transmission congestion and voltage instability caused by a large number of distributed power resources entering the grid, a rapid response demand response mechanism is required to quickly balance the power supply and demand in the grid. The OpenADR protocol is used between the power company and CEMS to conduct the related operations of demand response. CEMS can simultaneously manage multiple BEMSs, and there are two communication modes between CEMS and BEMS: OpenADR and TCP/IP. TCP/IP is used to transmit information about electricity consumption and electricity generation, while BEMS transmits instructions to the control server through Socket to control the Internet of Things device. CEMS and BEMS store and read data to and from the database through the defined API, while the control server is mainly responsible for writing the collected data to the database in real-time. The SCADA system includes a control server (CS), a switch, a TCP/remote terminal unit (RTU) converter (TRC), a PLC, and various Internet of Things devices, such as electricity meters and temperature and humidity sensors, as shown in Figure 2. The communication protocol used in the SCADA system is the Modbus protocol. CS is responsible for controlling the Internet of Things devices, and the network switch is responsible for enabling CS to control multiple Internet of Things devices, thus increasing the scalability of the system. TRC is responsible for the conversion between Modbus TCP and Modbus RTU in the Modbus protocol, while the Internet of Things devices are responsible for collecting various information, such as information about electricity consumption, temperature, humidity, etc., or executing other CS instructions. Managers can view system status or perform energy management services in a timely manner through Web/APP.

**Figure 3:**
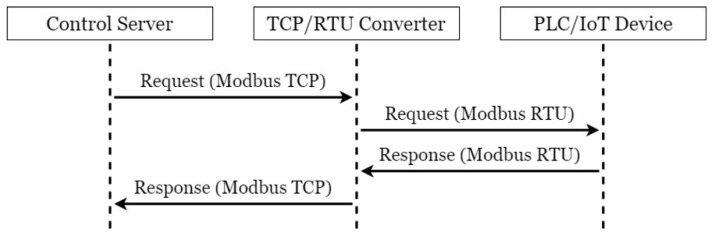
A simple communication flow of original devised system.

The communication process of messages and instructions in Figure 1 are shown in Figure 3. CS sends control requests to TRC at regular intervals through Modbus TCP or sends control instructions from BEMS to TRC. Upon receiving the instructions, TRC converts Modbus TCP to Modbus RTU and transmits it to the corresponding Internet of Things device. Upon receiving a request for information, the device immediately sends back the specified information in the form of Modbus RTU; for example, an electricity meter sends back the information of its several registers after receiving a request for electricity consumption information. Upon receiving a request to perform an operation, the device takes the corresponding action and sends a reply indicating the completion of the operation; for example, after receiving a request to start, a motor in a factory will start and send back a reply indicating the successful startup. After receiving the reply from the device, TRC converts it from Modbus RTU to Modbus TCP and sends it to CS, which processes this information and stores it in the database.

**Figure 4:**
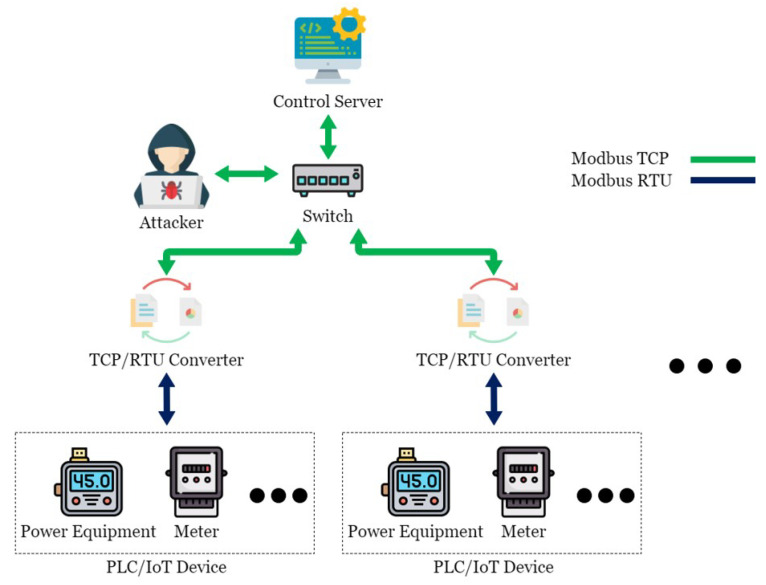
The scenario of Man-In-The-Middle attack.

**Figure 5:**
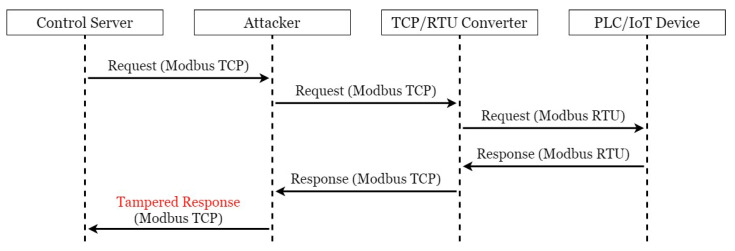
A simple attack process to original devised system.

There are many security vulnerabilities in the general Modbus protocol. This paper constructed an actual man-in-the-middle attack to attack this system, as shown in Figure 4. In order to achieve the purpose of tampering with the packet with the man-in-the-middle attack, an attacker was added to the original SCADA system architecture and connected to the network switch to allow it to enter the internal network and act as a man-in-the-middle between CS and TRC. The attack process took the request of CS to regularly send electricity consumption information to the electricity meter as an example, as shown in Figure 5. First, as CS was unaware of the presence of the attacker, it transmitted requests to TRC normally; at this point, the hacker used Address Resolution Protocol (ARP) Spoofing to intercept the packet between CS and TRC. In this situation, as the hacker did not need to tamper with the request packet, it was sent intact, and after receiving and converting the format, TRC sent it to the electricity meter. The electricity meter sent back various information on electricity consumption in the register as requested. TRC converted the format and sent it back to CS. At this time, the packet was intercepted by the hacker, and the electricity consumption information in the packet was tampered with. At the same time, the tampered packet was transmitted to CS, which processed the real-time information in this packet and stored it in the database. In the absence of any security protection mechanism, hackers can easily steal electricity without being detected by managers. This paper conducts experiments in this context to prove the existence of vulnerabilities and the feasibility of the proposed TTAS architecture.

## 4. TTAS: Trusted Token Authentication Service

**Figure 6:**
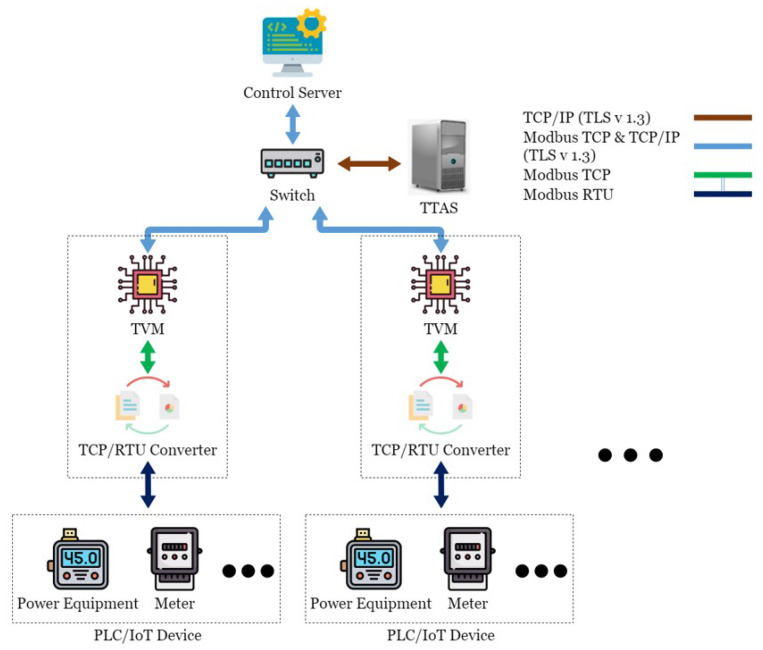
The proposed architecture.

In this paper, a server named Trusted Token Authentication Service (TTAS), which is responsible for generating tokens for legitimate devices, was added to the SCADA system architecture, as shown in Figure 6. The TTAS system includes a Trusted Verification Module (TVM) machine, which is mainly responsible for encrypting and decrypting packets and verifying device legitimacy. TVM and TRC are regarded as the same hardware device, while CS is additionally responsible for encrypting and decrypting packets and verifying device legitimacy. The connection among CS, TVM, and TTAS is encrypted through TLS 1.3 to establish a secure connection. Therefore, this paper proposes a solution called an encryption and verification mechanism, which combines two ways to solve this security problem. The first is to use Transport Layer Security 1.3 [33] to encrypt Modbus TCP; hackers cannot easily read or tamper with the content even if they intercept the packet. The second is to use tokens to verify CS and Internet of Things devices; hackers cannot use a reply attack to disable the SCADA system.

### 4.1. Authentication Mechanism

**Figure 7:**
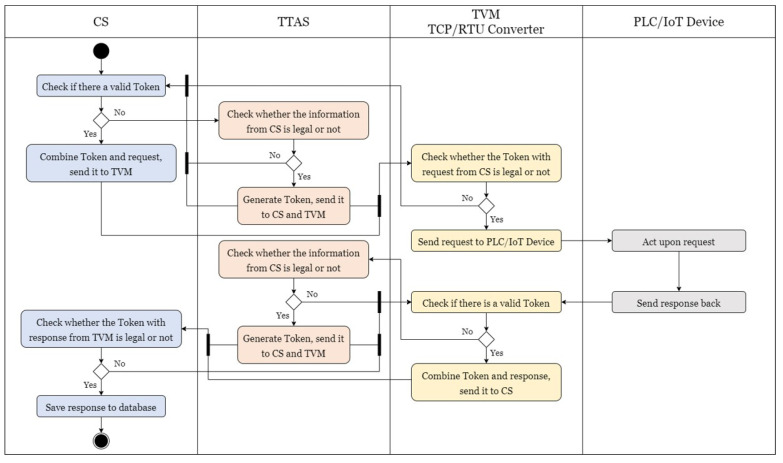
The authentication process.

**Figure 8:**
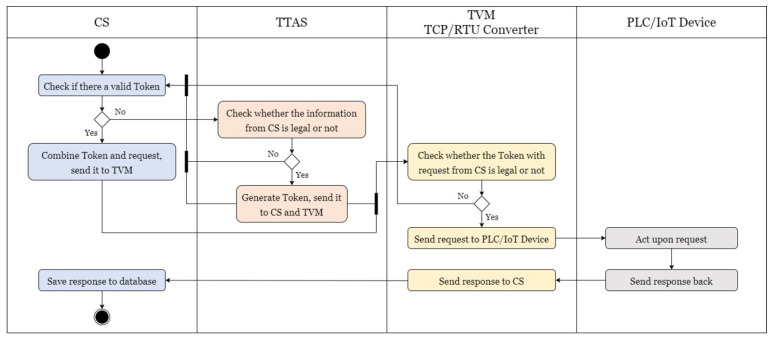
The simplified process of authentication.

First, before CS sends a timed request or a control request from BEMS, CS checks whether the token itself is legitimate. If there is no token or there is an illegitimate token, CS must apply to TTAS for a legitimate token. CS is required to provide TTAS with specified information when applying, and if there is a legitimate token, the token is combined with the request to be sent out and transmitted to TVM for subsequent actions. When receiving a token application from CS, TTAS checks the information provided by CS; if the information is legitimate, it generates a token and transmits it to CS and TVM simultaneously. If the information is illegitimate, the error information is returned to CS to reapply. After receiving a message from CS, TVM checks whether its token is consistent with the token from TTAS and is legitimate. If so, the request in the message is transmitted to the PLC/IoT device; otherwise, the error information is returned to CS to reapply for a token. After receiving the request, the PLC/IoT device immediately performs the specified action and sends a message back to TVM. Upon receiving the reply, TVM checks whether the token itself is legitimate, as does CS. If not, specified information is sent to TTAS to apply for a token; if so, the token is combined with the reply and returned to CS. After receiving the token application from TVM, TTAS performs the same process as CS for tokens. Finally, when CS receives a message from TVM, it first checks whether the token in it is consistent with a legitimate token from TTAS. If not, it rejects this message and sends an error message to TVM to reapply for a token; if so, the reply information in the message is stored in the database. The complete authentication mechanism process is shown in Figure 7, which checks whether there is a legitimate token in the request and reply processes, and such shortcomings lead to a delay in message reply. Therefore, this article also proposes a simplified authentication mechanism, as shown in Figure 8. When the PLC/IoT device transmits information to TVM, TVM does not check whether it has its own legitimate token, but immediately sends the reply to CS. CS does not check whether the reply has a legitimate token, but stores the information of the reply directly in the database. The disadvantage of this method is that it does not verify the identity of TVM, while the advantage is that it can effectively reduce the operation time of TVM and CS, as well as the utilization rate of the CPU and memory.

### 4.2. Generating Tokens

Tokenization [44] has been applied in many fields, such as network communication, information security, credit card, third-party verification, etc. Through tokenization technology, an IoT device can be mapped to a token, which is a reference (identifier) without external meaning or use value; thus, it is suitable for the protection of sensitive data, safe storage, audit, certification and authorization, and service. In the future, when other devices apply for tokens from TTAS, in order to obtain legitimate tokens, they must also send information to TTAS in accordance with this rule. After receiving an application, TTAS checks the seven information items, and if all of them meet the requirements, it generates a token and sends it back. If more than one item in the information does not meet the requirements, it sends back an error message for the purpose of reapplying. The different meanings of the token rules are described as follows:

- src_ip: the IP of the device applying for the token, i.e., the IP of the applicant;
- src_hostname: the hostname of the applicant;
- src_mac_addr: the mac address of the applicant;
- dst_ip: the IP of the object to be authenticated by the applicant, i.e., the IP of the verifier;
- dst_port: the socket port of the verifier;
- dst_hostname: the hostname of the verifier;
- dst_mac_addr: the mac address of the verifier.

A set of tokens is generated through RSA-2048 and SHA-256. The significance of the token format is, as follows:

- iss: the device for generating tokens, i.e., TTAS, represented here by the IP of TTAS;
- iat: the time when the token was generated;
- exp: the expiry date of the token, i.e., the time during which the token can exist legitimately;
- aud: the IP of the applicant;
- hostname: the hostname of the applicant;
- mac_addr: the mac address of the applicant;
- priority: the priority of the token;
- service_type: the type of the token, i.e., the token can be used in a variety of applications.

CS and TVM must check the legitimacy of the token before combining and transmitting the token with the request or reply, and after receiving the message including the token from the other party. The parts to be checked are (1) iss, (2) iat, (3) exp, (4) aud, (5) hostname, (6) mac_addr, and (7) service_type. If these seven items are all legitimate, the token is judged to be legitimate; otherwise, the token is judged to be illegitimate.

### 4.3. System of Encryption and Verification Mechanism

**Figure 9:**
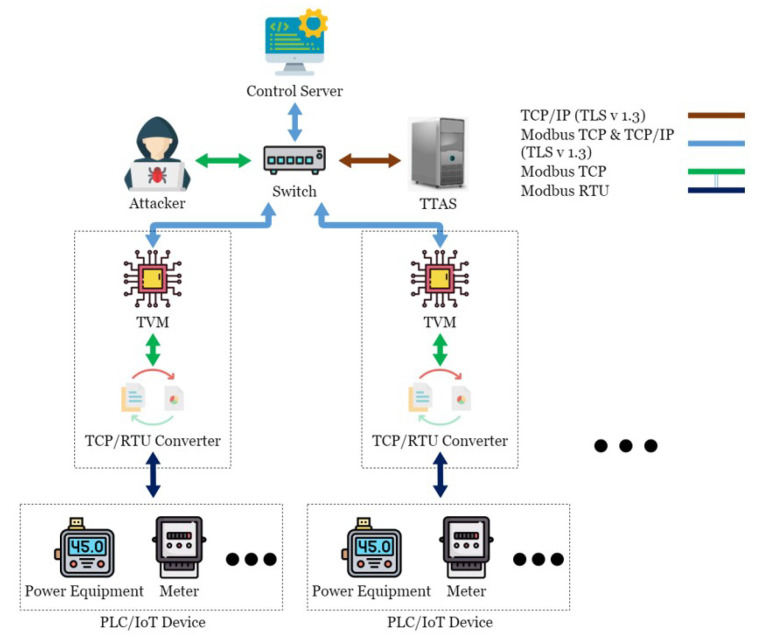
The simulation of attacking proposed system.

**Figure 10:**
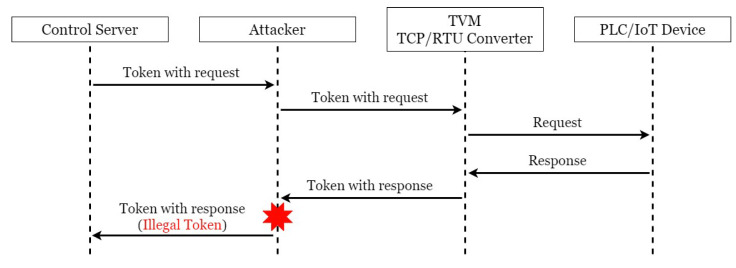
The solution of defense against attacking.

The man-in-the-middle attack was used to attempt to attack the solution proposed in this paper, as shown in Figure 9. Taking the request of the electricity consumption information of the electricity meter as an example, it was provided with the encryption and verification mechanism, and it was preset that CS and TVM already had legitimate tokens. The attack process is shown in Figure 10. CS combined the request to return the electricity consumption information with the token, which was sent to the electricity meter, and then the hacker used ARP spoofing to intercept the packet between CS and TVM. Similarly, the hacker sent the request intact, and after TVM checked the token in the message and confirmed that it was legitimate, it sent the request to the specified electricity meter, which replied to the electricity consumption information according to the request. TVM combined this reply with the token and sent it back to CS. While the packet would still be intercepted by the hacker, because the packet was encrypted, the hacker could not confirm the packet format,; thus, the hacker had only two options: one was to give up tampering with the packet, and the other was to modify the packet at will; the second way would tamper with the token and render it illegitimate. CS would find problems when checking and send an error message to TVM regarding the illegitimacy of the token in the message, and TVM would resend until timeout. The solution proposed in this paper can indeed solve this security vulnerability.

## 5. Security Analysis

In this section, the security of the encryption and verification mechanism, as proposed in this paper, was analyzed according to the methods suggested in [39,45,46,47]. The items were analyzed to determine whether the identity information of the Internet of Things devices is confidential; whether there is mutual authentication; and whether the proposed mechanism can resist a man-in-the-middle attack, a replay attack, and a impersonation attack.

### 5.1. Claim 1: The Identity Information of Internet of Things Devices Is Confidential

The identity of Internet of Things devices includes (1) converter_ip, (2) converter_port, (3) slave_id, (4) starting_address, and (5) quantity_of_x. Hackers must know the above five items of information to arbitrarily counterfeit CS or TVM, and accurately access the information of Internet of Things devices. As the mechanism proposed in this paper uses TLS 1.3 for encryption, hackers cannot obtain the identity of the Internet of Things devices when the packets are encrypted.

### 5.2. The Proposed Mechanism Provides Mutual Authentication

When CS sends the message including the request and the token to TVM, or when TVM sends the message including the reply and the token to CS, authentication is required. After receiving the message from CS, TVM checks the token in it; if the token is the same as the previous one from TTAS, and legitimate, it is judged to be authenticated. After receiving the message from TVM, CS also checks the token in it; if the token is the same as the previous one from TTAS, and legitimate, CS is considered to be authenticated. In addition, if hackers want to forge into valid CS or TVM, they must generate a valid message, that is, it must generate a legitimate token and combine it with the request/reply. However, because the token is generated by RSA-2048 and SHA-256, there is no way for hackers to understand the composition of the token, thus they cannot successfully attack the system.

### 5.3. The Proposed Mechanism Can Resist Man-in-the-Middle Attack

When CS sends the message including the token and the request to TVM, or when TVM sends the message including the token and the reply to CS, hackers can use ARP spoofing to intercept packets. However, as the transmission channel between CS and TVM has been encrypted with TLS 1.3, even if the packets are intercepted by hackers, they are still unable to tamper with the encrypted packets, meaning hackers can only arbitrarily tamper with the encrypted information. As CS and TVM check the legitimacy of the token after receiving the message, a packet that is arbitrarily tampered with is judged to be an illegitimate message.

### 5.4. The Proposed Mechanism Can Resist Replay Attack

Assume that CS has sent a message including the token and the request to TVM, if hackers attempt to impersonate a legitimate CS by retransmitting the same message, TVM rejects the message because the token uses timestamps, namely, “iat” and “exp”. In addition, assume that TVM has sent a message including the token and the reply to CS. Similarly, if hackers attempt to impersonate a legitimate TVM by retransmitting the same message, it is rejected because the token also contains timestamps; thus, any attempt by hackers to retransmit the message to CS or TVM is rejected. From another perspective, as the transmission channel has been encrypted with TLS 1.3, hackers cannot easily obtain a legitimate token to create the same or legitimate message.

### 5.5. The Proposed Mechanism Can Resist Impersonation Attack

Hackers use three types of fake identities: fake CS, fake TVM, and fake Internet of Things devices. In the first and second cases, hackers who want to impersonate the CS or TVM identity must provide the information shown in Table 1 to TTAS. However, as hackers do not know what kind of data is needed to apply to TTAS, they cannot apply for a legitimate token. Even if hackers know what information to provide to TTAS, they can only find out (1) src_ip, (2) src_mac_addr, (3) dst_ip, (4) dst_port, and (5) dst_mac_addr, but cannot know src_hostname or dst_hostname. Therefore, hackers cannot impersonate CS or TVM. In the third case, if hackers want to impersonate Internet of Things devices, they must know the relevant information of legitimate Internet of Things devices, as described in Section 5.1; thus, hackers cannot impersonate Internet of Things devices.

## 6. Experiment

This section will design a simulated experimental environment to illustrate the system architecture and mechanism proposed in this paper. In addition, the proposed system architecture is applied to an actual field to prove its feasibility and analyze its effectiveness.

### 6.1. Simulating Experimental Environment

**Figure 11:**
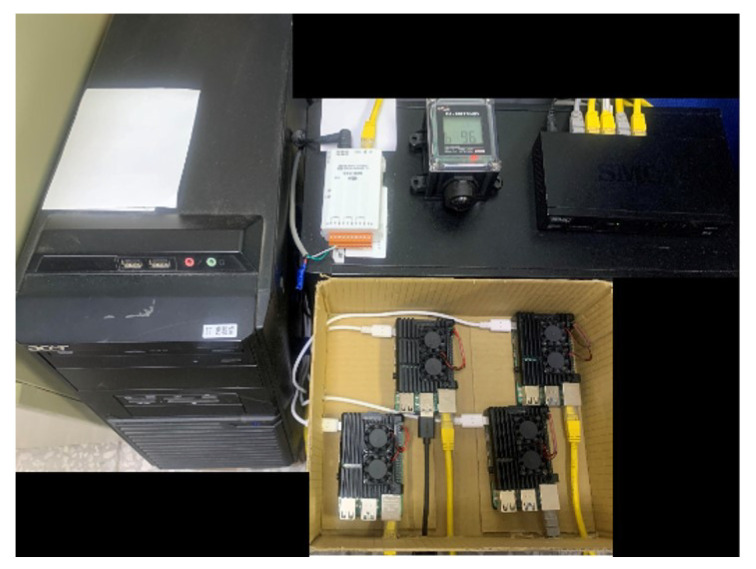
The simulation environment with encrypted validator.

In practice, a physical server is used as the control server to transmit requests to access Internet of Things devices. TTAS uses a Raspberry Pi to check the legitimacy of the control server and the Internet of Things devices, and generates a token according to the results. TVM uses a Raspberry Pi to run the verification module. Encrypted Validator uses a Raspberry Pi as the extension module of CS. The man-in-the-middle attack mode uses a Raspberry Pi simulation as the attack node. The detailed specifications are shown in Table 1 and Table 2. The TCP/RTU converter adopts the device of ICP DAS tGW-735, while the Internet of Things device adopts the temperature and humidity sensor of ICP DAS DL-100TM485. The simulation experimental environment is as shown in Figure 11.

### 6.2. Verifying the Encryption and Verification Mechanism

**Figure 12:**
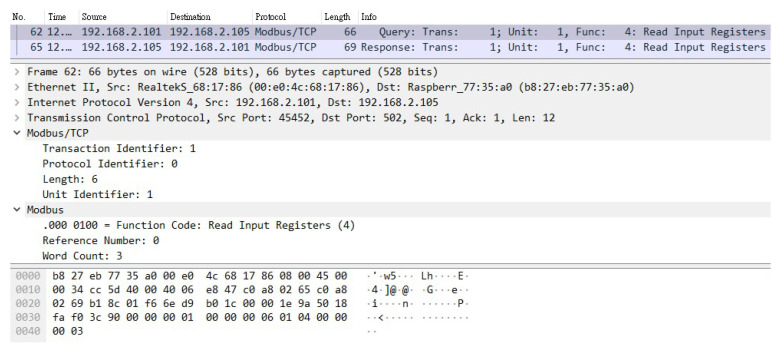
The unencrypted request packet.

**Figure 13:**
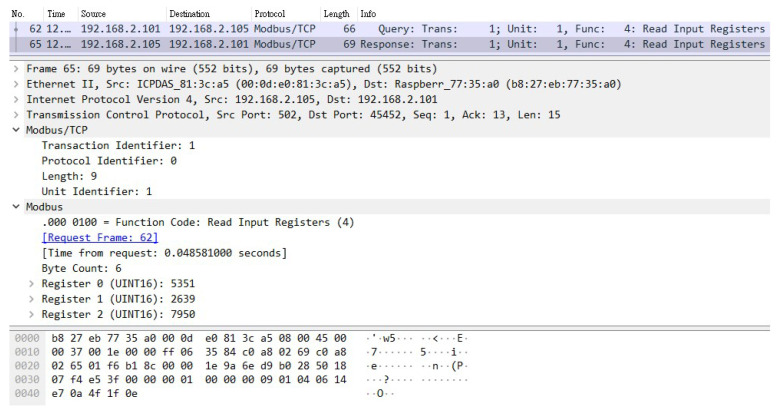
The unencrypted response packet.

The solution proposed in this paper regarding the security vulnerabilities of the SCADA system using the Modbus protocol must achieve two objectives: (1) effectively prevent man-in-the-middle attack after adding the encryption and verification mechanism, and (2) avoid excessive overhead after adding this mechanism. The Internet of Things device used in this experiment is a temperature and humidity sensor. Figure 12 and Figure 13 show the packets captured by CS, which are the request and reply packets between CS and TVM, respectively. It can be seen from these two figures that the transmission protocol used for both request and reply pockets is Modbus TCP. Moreover, it is clear that the information inside the packets is not encrypted, and all content is presented in plaintext.

**Figure 14:**
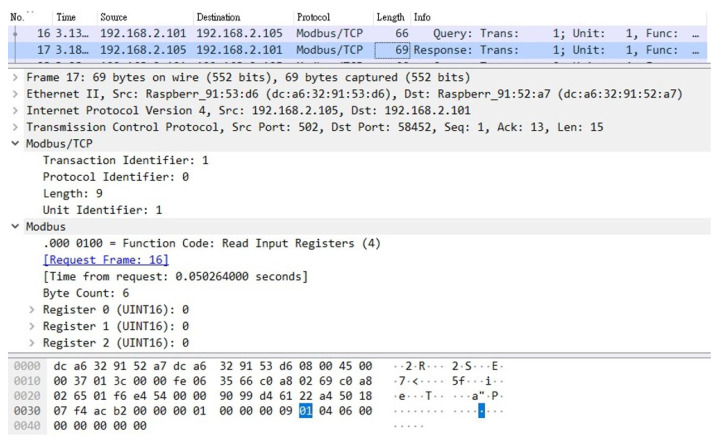
The tampered packet.

A Raspberry Pi was added to this SCADA system as an attacker, which was connected to a switch to enable it to actually enter the system. First, the attacker guided the packets between CS and TVM to the device through ARP spoofing. In this way, the unencrypted request and reply packets similar to those shown in Figure 12 and Figure 13 can be obtained. The attacker used the program to tamper with these stolen packets and then send them out (changing the value to 0), as shown in Figure 14. This is a simple physical man-in-the-middle attack that can damage the system or steal resources, such as stealing electricity.

**Figure 15:**
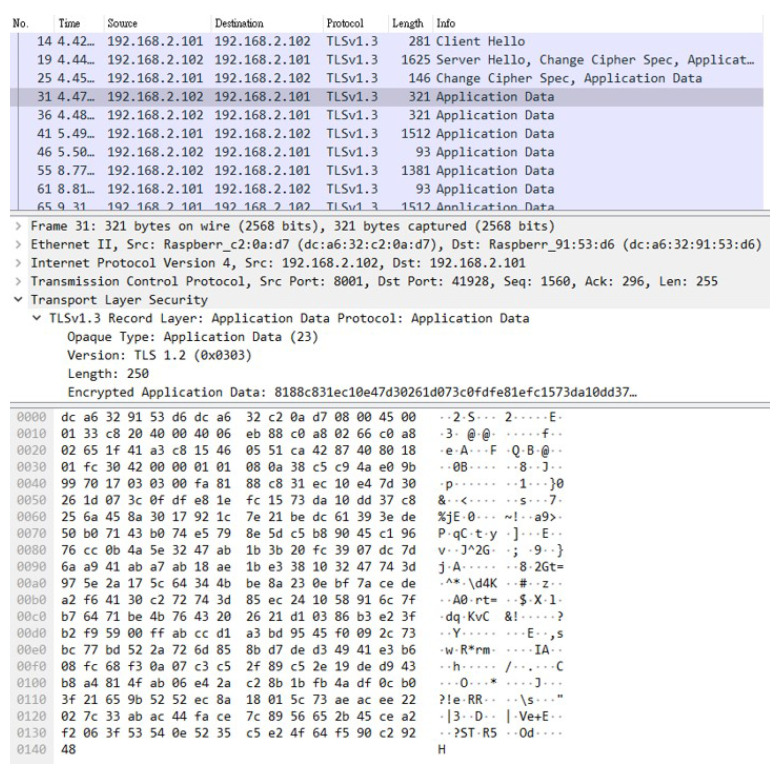
The packet is encrypted by TLS 1.3.

From the above experiments, it can be proved that there are indeed security vulnerabilities in the SCADA system using the Modbus protocol. Therefore, in order to verify the feasibility and effectiveness of using the encryption and verification mechanism in the SCADA system, the following two items must be achieved, respectively: (1) encrypt all packets using TCP and the Modbus TCP protocol among CS, TTAS, and TVM, and (2) use tokens generated by TTAS to authenticate CS and TVM with each other. As shown in Figure 15, all packets have been encrypted with TLS 1.3; therefore, unless the attacker succeeds in cracking the currently widely used TLS 1.3, the contents of these packets cannot be easily parsed by the attacker even if they are stolen. Thus, it can be proved that using TLS 1.3 to encrypt packets can effectively prevent hackers from tampering with packet contents.

Unfortunately, if only TLS 1.3 is used to encrypt packets in the system, attackers can still attempt to communicate with CS by impersonating a legitimate TVM, thereby causing damage to the system. Therefore, it is necessary to verify the identity of the device to ensure security. CS and TVM apply for a token from TTAS by providing specified information and use the legitimate token for two-way authentication between each other. The message returned by TVM includes the legitimate token to be checked by CS, and the sensor value in the message is read. This is half of the authentication process. The other half of authentication occurs when CS sends a message to TVM, which also checks the token in the message, in order to fulfill two-way authentication. This method can effectively prevent unauthenticated devices from entering the system and, thus, prevent hackers from using various attacks, such as impersonation attacks, replay attacks, etc., to attack the system.

### 6.3. Actual Field Experiment Results

**Figure 16:**
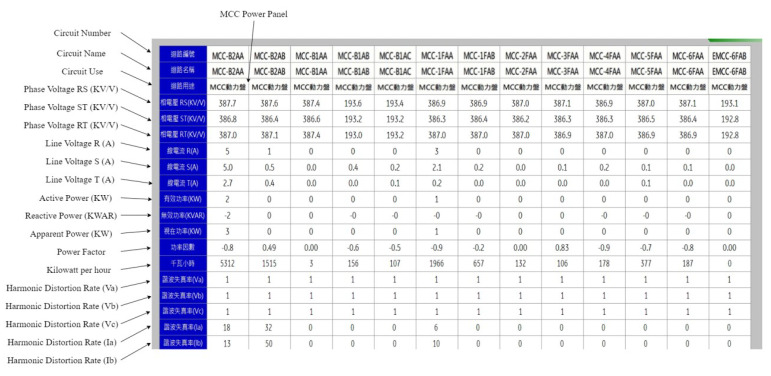
The real-time information of electricity meters in EMS.

**Figure 17:**
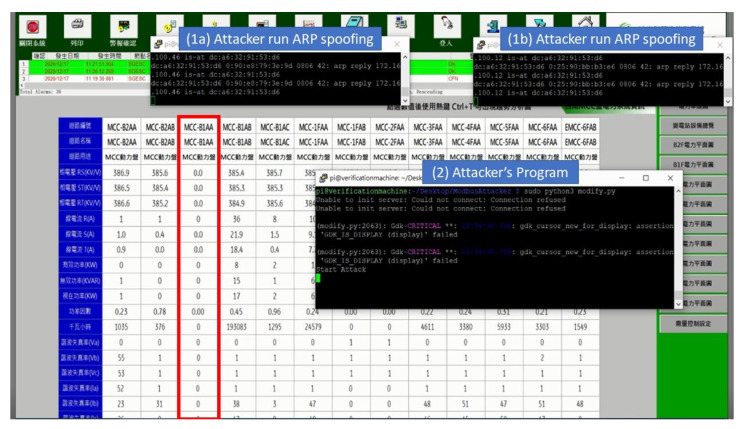
The attack process on electricity meter without protection mechanism.

In the energy management system used by the Green Energy Science City in southern Taiwan, the communication protocol used in the SCADA system is the Modbus protocol, which lacks any protective measures. Figure 16 shows the real-time information of some electricity meters in an EMS area. It can be seen from Figure 16 that the construction of this field has been completed at present, meaning all electricity meter values can be read normally; thus, the electricity consumption data of all devices are monitored. This paper used the actual field to prove the following points: (1) the SCADA system using the Modbus protocol does have security vulnerabilities, (2) running the encryption and verification mechanism proposed in this paper does not affect the original system, and (3) the encryption and verification mechanism can effectively protect against this security vulnerability. Figure 17 shows the actual attack on a specific electricity meter in this field; subfigures (1a) and (1b) mean stealing packets between CS and TVM by using ARP spoofing. Subfigure (2) means the procedure to execute tampering of packet contents. It can be seen from Figure 17 that the values of the electricity meter (red box) with circuit number MCC-B1AA have all been tampered with, and returned to 0. In addition, CS in this system does not check whether the received values are abnormal, meaning even if changed to 0, they do not jump out of the alert, but are stored directly in the database. This experiment proves that without an effective security protection mechanism, hackers can easily steal electricity.

**Figure 18:**
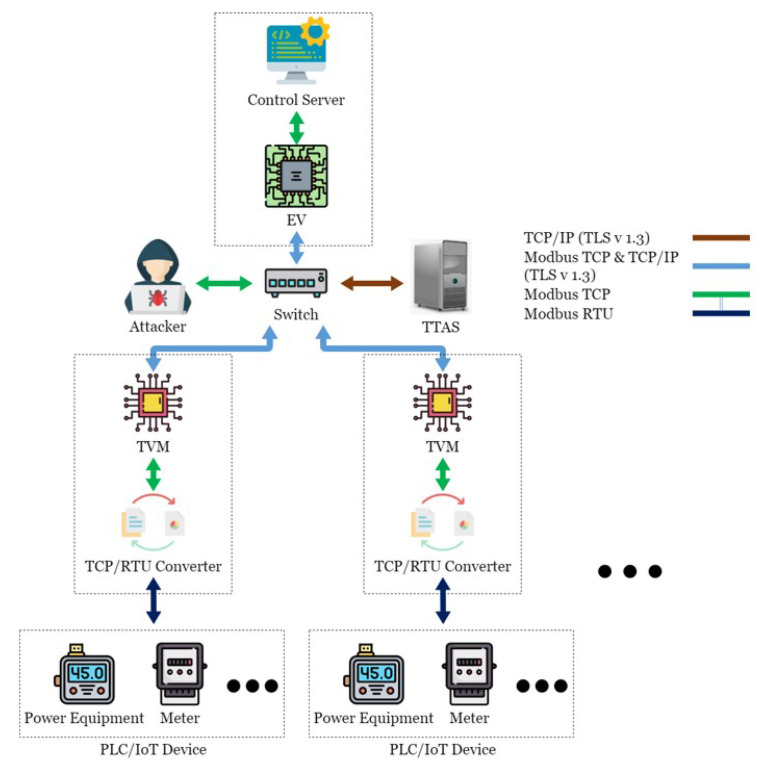
The proposed system with encrypted validator.

In order to run the encryption and verification mechanism proposed in this paper without affecting the original system, an additional Encrypted Validator (EV) was added to the SCADA system, which was connected between CS and the switch, and its task was to run the encryption and verification mechanism instead of CS, as shown in Figure 18. The authors of [48] mentioned that introducing a Fog End-Device into the existing system architecture is a good strategy to strengthen the security of the Internet of Things. This paper proposes a security mechanism to be implemented on the Raspberry Pi device, while avoiding affecting the existing system. The existing system security can be upgraded immediately without stopping the system operation. In terms of communication protocols, such as Modbus TCP, Modbus RTU, it can also be run directly without any modification after the upgrade is completed.

**Figure 19:**
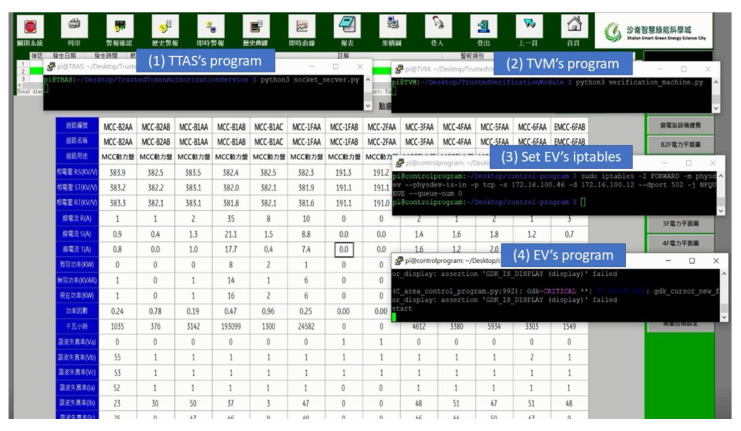
Add the encrypted validator on electricity meter.

**Figure 20:**
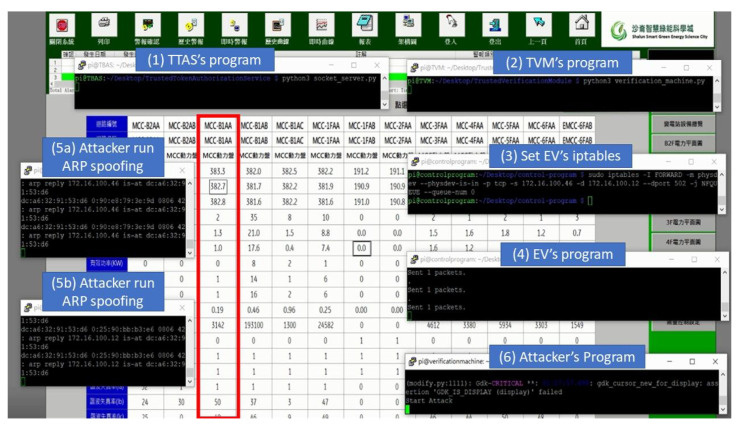
The attack process on electricity meter with proposed system.

Figure 19 shows running the encryption and verification mechanism in this field. The steps to run this mechanism are (1) execute the procedure of TTAS, meaning that CS and TVM can apply for tokens from them; (2) execute the procedure of TVM, meaning that EV can run the encryption and verification mechanism with it; (3) set iptables for EV to run in transparent mode; and (4) execute the procedure of EV, meaning that TVM can run the encryption and verification mechanism with it. As can be seen from Figure 18, after the encryption and verification mechanism is successfully executed, the system can still read the electricity meter value normally. This experiment proves that there is no problem or system crash caused by running the encryption and verification mechanism. Figure 20 shows the actual operation of the encryption and verification mechanism in this field, which attempts to attack the system with the same tampering attack as described previously. However, it can be clearly seen from Figure 18 that the value of the electricity meter with circuit number MCC-B1AA has not been tampered with into an abnormal value, and all the values are displayed normally. The experiment proves that the system can effectively prevent hackers from physical man-in-the-middle attack after running the encryption and verification mechanism proposed in this paper.

### 6.4. Efficiency Analysis

**Figure 21:**
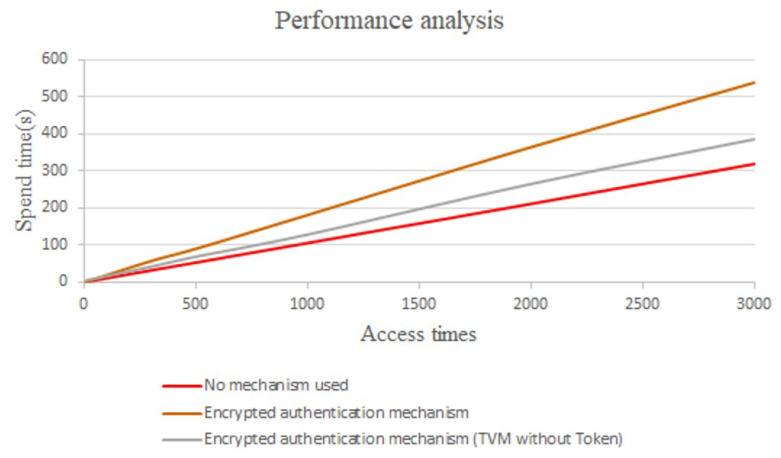
The performance analysis.

Figure 21 shows the efficiency analysis of CS accessing a single Internet of Things device, where the horizontal axis is the number of times that CS accessed the Internet of Things device, while the vertical axis is the time spent on such access. The red line denotes that the encryption and verification mechanism was not executed, the gray line denotes that the simplified encryption and verification mechanism was executed, and the brown line denotes that the complete encryption and verification mechanism was executed. It can be seen from Figure 21 that the time consumed by running the simplified encryption and verification mechanism is about 1.2 times that of not using the encryption and verification mechanism, while the time consumed by running the complete encryption and verification mechanism is about 1.7 times that of not using the encryption and verification mechanism. Although from the data point of view, it takes a lot of time, it actually takes a very short time without running the encryption and verification mechanism. However, in practice, as the SCADA system lacks strict requirements regarding the access time of most Internet of Things devices, it basically only requires that a reply is received from Internet of Things devices within 1–2 s. Furthermore, we learned from the system integrator that the average time from transmission to response must be controlled within 1 s when integrating this mechanism. It can be seen from Figure 21 that 500 accesses take less than 100 s. The average delay times for executing the complete mechanism and simplified mechanism are 0.179 s and 0.128 s, respectively. According to expert’s recommendation, the proposed architecture is in line with the demand. Therefore, the mechanism proposed in this paper has good efficiency in the actual field.

## 7. Conclusions

This paper proposes an encryption and verification mechanism based on the trusted token authentication service and Transport Layer Security (TLS) protocol to ensure SCADA network security and prevent attackers from physical attacks in the energy management system of Industrial Internet of Things. The device and remote control server can complete two-way authentication through a token, as the token has the characteristic of high privacy, which can prevent sensitive information from being easily disclosed. The mechanism proposed in this paper can protect an industrial network from external threats and execute an authentication process before allowing any entity access to network resources. This study deployed and verified the proposed protection mechanism in the energy management system in the Green Energy Science City in southern Taiwan. According to the experimental results, the security defense architecture proposed in this paper can effectively improve security and is compatible with the actual field system. In addition, as the protection mechanism proposed in this paper has very low hardware costs, it is helpful for large-scale deployment and implementation in the actual case field. However, the main limitation of this paper is that the proposed architecture cannot change the existing power management system and network deployment architecture. The authentication mechanism requires the encrypted validator proposed in this paper and needs to be connected between the control server and the switch. This adds additional development burden. In the future work, the work of the encrypted validator will be gradually integrated into the control server and provide more comprehensive authentication and management functions. Moreover, it is expected that TVM and TRC can be integrated into a single hardware device in the future, thus reducing transmission delays and security risks.

## Figures and Tables

**Table 1:** The server specification.

	Control Server	TTAS
CPU	Intel Core i7 3770 @ 3.40 GHz	Broadcom BCM2711,
		Quad core Cortex-
		-A72 64-bit SoC @ 1.5 GHz
RAM	24 GB DDR3 @ 1600 MHz	4 GB LPDDR4-3200 SDRAM
Storage	256 GB SSD 512 GB HDD	64 GB
Ethernet	1 Gb/s	1 Gb/s
OS	Windows 10	Raspbian 10 (buster)
Kernel Version	10.0.18363.1316	5.4.72-v71+

**Table 2:** The edge node specification.

	Trusted Verification Module	Encrypted Validator/Attacker
CPU	Broadcom BCM2711,	Broadcom BCM2711,
	Quad core Cortex-	Quad core Cortex-
	-A72 64-bit SoC @ 1.5 GHz	-A72 64-bit SoC @ 1.5 GHz
RAM	8 GB LPDDR4-3200 SDRAM	4 GB LPDDR4-3200 SDRAM
Storage	64 GB	64 GB
Ethernet	1 Gb/s	1 Gb/s
OS	Raspbian 10 (buster)	Raspbian 10 (buster)
Kernel Version	5.4.72-v71+	5.4.72-v71+

## References

[1] Wollschlaeger M., Sauter T., Jasperneite J. (2017). The Future of Industrial Communication: Automation Networks in the Era of the Internet of Things and Industry 4.0. IEEE Ind. Electron. Mag..

[2] Jeschke S., Brecher C., Meisen T., Özdemir D., Eschert T. (2017). Industrial internet of things and cyber manufacturing systems. Industrial Internet of Things.

[3] Schwab K. (2017). The Fourth Industrial Revolution.

[4] Stouffer K., Pillitteri V., Lightman S., Abrams M., Hahn A. (2014). Guide to Industrial Control Systems (ICS) Security.

[5] Boyer S.A. (2009). Supervisory Control and Data Acquisition.

[6] Webb J.W., Reis R.A. (2002). Programmable Logic Controllers Principles and Applications.

[7] Bobat A., Gezgin T., Aslan H. (2015). The SCADA system applications in management of Yuvacik Dam and Reservoir. Desalin. Water Treat..

[8] Adnan S., Zheng S., Rouse M.D., Lu W., Opel K.C. (2003). Distributed Control System. U.S. Patent.

[9] Patel N.R., Risbeck M.J., Rawlings J.B., Wenzel M.J., Turney R.D. Distributed economic model predictive control for large-scale building temperature regulation. Proceedings of the American Control Conference.

[10] Clarke G., Reynders D., Wright E. (2004). Practical Modern SCADA Protocols: DNP3, 60870.5 and Related Systems.

[11] OPC Unified Architecture Specification. https://opcfoundation.org/developer-tools/specifications-unified-architecture.

[12] MQTT 5 Specification. https://docs.oasis-open.org/mqtt/mqtt/v5.0/mqtt-v5.0.html.

[13] Francino P.N., Huff C. (2016). Energy Management System. U.S. Patent.

[14] Miwa K. (2016). Building Energy Management System. U.S. Patent.

[15] Rotger-Griful S., Welling U., Jacobsen R.H. (2017). Implementation of a building energy management system for residential demand response. Microprocess. Microsyst..

[16] Horst G.R., Zhang J., Syvokozov A.D. (2009). Total Home Energy Management System. U.S. Patent.

[17] Al-Ali A.R., Zualkernan I.A., Rashid M., Gupta R., Alikarar M. (2017). A smart home energy management system using IoT and big data analytics approach. IEEE Trans. Consum. Electron..

[18] Liang W., Li K., Long J., Kui X., Zomaya A.Y. (2020). An Industrial Network Intrusion Detection Algorithm Based on Multifeature Data Clustering Optimization Model. IEEE Trans. Ind. Inform..

[19] Jokar P., Leung V.C.M. (2016). Intrusion Detection and Prevention for ZigBee-Based Home Area Networks in Smart Grids. IEEE Trans. Smart Grid.

[20] Conti M., Dragoni N., Lesyk V. (2016). A Survey of Man In The Middle Attacks. IEEE Commun. Surv. Tutor..

[21] Upadhyay D., Sampalli S. (2020). SCADA (Supervisory Control and Data Acquisition) systems: Vulnerability assessment and security recommendations. Comput. Secur..

[22] Radoglou Grammatikis P., Sarigiannidis P., Efstathopoulos G., Panaousis E. (2020). ARIES: A Novel Multivariate Intrusion Detection System for Smart Grid. Sensors.

[23] González I., Calderón A.J., Portalo J.M. (2021). Innovative Multi-Layered Architecture for Heterogeneous Automation and Monitoring Systems: Application Case of a Photovoltaic Smart Microgrid. Sustainability.

[24] Abad C.L., Bonilla R.I. An analysis on the schemes for detecting and preventing arp cache poisoning attacks. Proceedings of the 27th International Conference on Distributed Computing Systems Workshops (ICDCSW’07).

[25] Adams C. (2011). Encyclopedia of Cryptography and Security.

[26] Knowles W., Prince D., Hutchison D., Disso J.F.P., Jones K. (2015). A survey of cyber security management in industrial control systems. Int. J. Crit. Infrastruct. Prot..

[27] Volkova A., Niedermeier M., Basmadjian R., de Meer H. (2019). Security Challenges in Control Network Protocols: A Survey. IEEE Commun. Surv. Tutor..

[28] Ghosh S., Sampalli S. (2019). A Survey of Security in SCADA Networks: Current Issues and Future Challenges. IEEE Access.

[29] Ferst M.K., de Figueiredo H.F., Lopes J. Implementation of Secure Communication With Modbus and Transport Layer Security protocols. Proceedings of the 2018 13th IEEE International Conference on Industry Applications (INDUSCON).

[30] Figueroa-Lorenzo S., Añorga J., Arrizabalaga S. (2019). A Role-Based Access Control Model in Modbus SCADA Systems. A Centralized Model Approach. Sensors.

[31] Tidrea A., Korodi A., Silea I. (2019). Cryptographic Considerations for Automation and SCADA Systems Using Trusted Platform Modules. Sensors.

[32] Pricop E., Fattahi J., Parashiv N., Zamfir F., Ghayoula E. Method for authentication of sensors connected on modbus tcp. Proceedings of the 2017 4th International Conference on Control, Decision and Information Technologies (CoDIT).

[33] Rescorla E. The Transport Layer Security (TLS) Protocol Version 1.3. https://tools.ietf.org/html/rfc8446.

[34] El-Hajj M., Fadlallah A., Chamoun M., Serhrouchni A. (2019). A survey of internet of things (IoT) Authentication schemes. Sensors.

[35] Aman M.N., Chua K.C., Sikdar B. (2017). Mutual Authentication in IoT Systems Using Physical Unclonable Functions. IEEE Internet Things J..

[36] Qureshi M.A., Munir A. PUF-IPA: A PUF-based Identity Preserving Protocol for Internet of Things Authentication. Proceedings of the 2020 IEEE 17th Annual Consumer Communications and Networking Conference (CCNC).

[37] Zhang J.L., Qu G. (2019). Physical Unclonable Function-based Key-Sharing via Machine Learning for IoT Security. IEEE Trans. Ind. Electron..

[38] Choudhary K., Gaba G.S., Butun I., Kumar P. (2020). MAKE-IT—A Lightweight Mutual Authentication and Key Exchange Protocol for Industrial Internet of Things. Sensors.

[39] Esfahani A., Mantas G., Matischek R., Saghezchi F.B., Rodriguez J., Bicaku A., Maksuti S., Tauber M.G., Schmittner C., Bastos J. (2017). A Lightweight Authentication Mechanism for M2M Communications in Industrial IoT Environment. IEEE Internet Things J..

[40] Dammak M., Boudia R.R.M., Messous M.A., Senouci S.M., Gransart C. Token- based lightweight authentication to secure iot networks. Proceedings of the 2019 16th IEEE Annual Consumer Communications and Networking Conference (CCNC).

[41] Sari A., Lekidis A., Butun I. (2020). Industrial Networks and IIoT: Now and Future Trends. Industrial IoT.

[42] OpenMUC User Guide. https://www.openmuc.org/openmuc/user-guide/.

[43] Watson D., Piette M., Sezgen O. Machine to machine (M2M) technology in demand responsive commercial buildings. Proceedings of the 2004 ACEEE Summer Study on Energy Efficiency in Buildings.

[44] Nxumalo Z.C., Tarwireyi P., Adigun M.O. Towards privacy with tokenization as a service. Proceedings of the 2014 IEEE 6th International Conference on Adaptive Science and Technology (ICAST).

[45] Wen F., Li X. (2012). An improved dynamic id-based remote user authentication with key agreement scheme. Comput. Electr. Eng..

[46] Hsiang H.-C., Shih W.-K. (2009). Improvement of the secure dynamic ID based remote user authentication scheme for multi-server environment. Comput. Stand. Interfaces.

[47] Liao Y.-P., Wang S.-S. (2009). A secure dynamic ID based remote user authentication scheme for multi-server environment. Comput. Stand. Interfaces.

[48] Butun I., Sari A., Österberg P. (2020). Hardware Security of Fog End-Devices for the Internet of Things. Sensors.

